# Supplementary material for: Patient satisfaction with the quality of nursing care in critical care units and medical wards in West Bank Hospitals, Palestine: A cross-sectional study
Source: PLoS One. 2025 Apr 29;20(4):e0322804. doi: 10.1371/journal.pone.0322804 (PMC12040242; doi:10.1371/journal.pone.0322804)
Supplement: S1 File — These supplementary materials enhance the interpretation of patient satisfaction dynamics and support evidence-based recommendations for optimizing nursing care quality in critical care units and medical wards across West Bank hospitals. (DOCX) [file pone.0322804.s001.docx]

**Supporting Information**

S1 Table. Participant Demographics

• Age
 – 20–29 years: 74 (36.8%)
 – 30–39 years: 58 (28.9%)
 – 40–50 years: 24 (11.9%)
 – Above 50 years: 45 (22.4%)

• Gender
 – Male: 99 (49.3%)
 – Female: 102 (50.7%)

• Marital Status
 – Married: 150 (74.6%)
 – Unmarried: 51 (25.4%)

• Hospital Admission Source
 – Emergency Department: 102 (50.7%)
 – Transferred from Another Facility: 25 (12.4%)

S2 Table. Hospital Admission and Patient Health

• Number of Hospital Stays
 – Only once: 84 (41.8%)
 – Twice: 40 (19.9%)
 – Three times: 28 (13.9%)
 – Four times: 20 (10.0%)
 – More than four times: 29 (14.4%)

• Health Status
 – Very Poor: 21 (10.4%)
 – Poor: 30 (14.9%)
 – Fair: 71 (35.3%)
 – Good: 49 (24.4%)
 – Excellent: 30 (14.9%)

S3 Table. PSNCQQ Scores and Predictors of Satisfaction

• PSNCQQ Score Range: 19.0–95.0
• Mean PSNCQQ Score: 64.50 ± 14.16
• Overall Perceptions Mean Score: 9.65 ± 2.80
• Significant Predictors:
 – Age: B = 0.162, p = 0.005
 – Patient Health: B = 0.242, p = 0.001
 – Recent Hospital Stays: B = 0.107, p = 0.022

S4 Text. Ethical Approval Documentation

• Project Title: Patient Satisfaction of the Quality of Nursing Care in Intensive Care Units and Medical Wards in South of West Bank Hospital, Palestine
• Project Number: CAMS/CCNA/2/124
• Approval Date: January 9, 2024

S5 Methods. Statistical Analysis Details

• Software Used: IBM SPSS Statistics Version 26
• Analyses Conducted: Descriptive statistics, t-tests, one-way ANOVA, multiple linear regression
• Significance Threshold: P < 0.05

S6 Table. Comparison of Patient Satisfaction between ICU and Medical Ward Patients

• ICU Patients – N: 93; Mean (SD): 67.23 (13.98); t-value: 2.45; p-value: 0.015*
• Medical Ward Patients – N: 108; Mean (SD): 61.87 (14.05)
