# Supplementary material for: Patient satisfaction with the quality of nursing care in critical care units and medical wards in West Bank Hospitals, Palestine: A cross-sectional study
Source: PLoS One. 2025 Apr 29;20(4):e0322804. doi: 10.1371/journal.pone.0322804 (PMC12040242; doi:10.1371/journal.pone.0322804)
Supplement: Attachment — Submitted filename: Response to Reviewers.pdf [file pone.0322804.s003.pdf]

Point-by-Point (PBP) Table based on the provided editor comments:

Item	Comments	Response
**Title and Abstract**	The title reflects the study's scope well, but the abstract is dense. Consider reducing jargon and enhancing clarity for a broader audience. Ensure the abstract explicitly mentions: study objectives, methods, key findings, and implications.	Thank you for the suggestion. The abstract has been revised to reduce jargon and enhance clarity. Study objectives, methods, key findings, and implications are now explicitly stated.
**Introduction**	The introduction effectively outlines the context but could be more concise. Reduce background details that may be extraneous to the study focus.	Thank you for your feedback. The introduction has been condensed by removing extraneous background details while retaining the core context and focus.
**Methods**	Clearly define inclusion/exclusion criteria for participants. Provide more detail on how the PSNCQQ was adapted or validated for the Palestinian context.	We appreciate this suggestion. Inclusion and exclusion criteria have been clearly defined, and additional details on the adaptation and validation of the PSNCQQ for the Palestinian context have been included.
**Results**	Tables are clear but consider consolidating demographic and hospital admission data to improve readability. Add insights on why significant predictors (age, health status) influenced satisfaction and implications.	Thank you for your input. Demographic and hospital admission data tables have been consolidated for readability. Insights on significant predictors and their implications have been included.
**Discussion**	Expand on the global relevance of findings. How do the results compare with similar studies in other settings? Discuss limitations more comprehensively and suggest how future research can address them.	Thank you for your valuable feedback. The global relevance of the findings has been elaborated, and comparisons with similar studies in other settings have been included. Limitations have been discussed more comprehensively with suggestions for future research.
**References**	Ensure consistency in formatting as per PLOS ONE guidelines (e.g., DOI inclusion). Verify that all references are cited within the text.	Thank you for the reminder. References have been reformatted to comply with PLOS ONE guidelines, and all in-text citations have been verified for consistency and accuracy.
**Figures and Tables**	Tables are informative, but	Thank you for your suggestion.

	captions should briefly describe the content and purpose of the table. Ensure all tables and figures comply with PLOS ONE formatting standards.	Captions have been revised to describe the content and purpose of each table. All tables now comply with PLOS ONE formatting standards.
**Ethics Statement**	It is included and addresses ethical approval and consent. Ensure IRB approval details match the uploaded "IRB.pdf."	Thank you for the note. The ethics statement has been reviewed, and the IRB approval details have been verified to align with the uploaded "IRB.pdf."
**Financial and Competing Interests**	The manuscript states no specific funding and no competing interests. Confirm this aligns with the provided "Financial Disclosure" section.	Thank you. The financial and competing interests have been confirmed to align with the "Financial Disclosure" section.
**Data Availability**	The manuscript indicates all relevant data are included. Confirm this aligns with PLOS ONE's requirement for unrestricted data availability. If supplementary materials exist, ensure they are uploaded.	We appreciate this comment. The manuscript's data availability statement has been confirmed to align with PLOS ONE requirements, and any supplementary materials have been uploaded.
**Study Design**	The cross-sectional design is described appropriately. Add more detail on the selection of participating hospitals and potential sampling biases.	Thank you for the suggestion. Details on the selection of participating hospitals and potential sampling biases have been added to the study design section.
**Supplementary Materials**	Verify that the "STROBE checklist" and supporting files comply with the guidelines.	Thank you. The "STROBE checklist" and all supporting files have been reviewed and verified for compliance with the guidelines.
**Recommendations for Authors**	Enhance focus on actionable recommendations in the conclusion. Use simpler language to broaden accessibility while maintaining academic rigor. Confirm adherence to formatting and structure guidelines for PLOS ONE.	Thank you for the feedback. Actionable recommendations have been added to the conclusion. The language has been simplified for broader accessibility, and formatting has been revised to adhere to PLOS ONE guidelines.

Reviewer #1 – Point-by-Point (PBP) Response

Item	Comments	Response
**Title**	The title needs modification. No need to mention the south of Palestine.	Thank you for the suggestion. The title has been revised, and references to "south of Palestine" have been

		removed.
**Abstract**	Needs restructuring and better organization. Abbreviations like ICU and CCU are used only onc, spell them out. Ensure consistency in describing hospitals (governmental vs. non-governmental). Methods should mention statistical tests, not just SPSS. The results should be concise, and the conclusion should align with significant findings.	The abstract has been restructured for clarity. ICU, CCU, and MDW are now spelled out. Consistency regarding hospital types has been ensured. Statistical tests (t-tests, ANOVA, regression) are explicitly mentioned. The results section has been made more concise, and the conclusion now reflects key recommendations.
**Introduction**	Discuss the quality of nursing care in-depth from an international and local perspective. Clarify the study's purpose and explain why ICU and medical wards were selected.	The introduction has been expanded to include a more in-depth discussion of nursing care quality globally and locally. The study’s purpose is now clearly stated, and the rationale for selecting ICU and medical wards has been explained.
**Methods**	Remove "quantitative" from the study design. No need to mention the advantages of the design. Ensure consistency in the study setting. In sample size calculation, include all components. Clarify inclusion and exclusion criteria. Provide details on the scoring system of the tool. If the Arabic version was used, mention it and its validity/reliability. Do not mention that it was not validated in Palestine, calculate Cronbach’s alpha. Add normality assessment and regression assumptions.	"Quantitative" has been removed. The study setting is consistently described. Sample size calculation components are now detailed. Inclusion and exclusion criteria are clearly outlined. The scoring system of the tool is now explained. The use of the Arabic version is mentioned, along with validity (Cronbach’s alpha = 0.91). Normality assessment and regression assumptions (Kolmogorov–Smirnov test, multicollinearity check) have been included.
**Results**	Focus on significant results. Consider removing sociodemographic comparisons unless highly relevant. If comparing ICU vs. medical ward patients, structure it properly. Ensure regression assumptions (normality, correlation, etc.) are applied.	Thank you for your valuable feedback regarding the inclusion of sociodemographic differences in the results section. While we understand the concern about streamlining the findings, we believe that retaining these analyses is essential for ensuring a more rigorous and comprehensive interpretation of patient satisfaction. Sociodemographic variables, including age, gender, marital status, and health status, have been widely recognized in previous literature as influential factors in shaping patient experiences and

		satisfaction with healthcare services. By maintaining these analyses, we ensure that the study's findings are contextualized within a broader demographic framework, allowing for a deeper understanding of the variability in patient satisfaction across different groups. Moreover, retaining the sociodemographic table enhances the transparency and replicability of our research. It provides readers with a detailed view of the sample characteristics, ensuring that any observed differences in patient satisfaction are interpreted in relation to relevant background variables. Additionally, our regression analysis demonstrated that certain demographic variables, such as age and health status, were significant predictors of satisfaction. Removing these results and tables could lead to an incomplete representation of the factors influencing patient perceptions of nursing care. Given the importance of these demographic insights, we respectfully propose retaining the sociodemographic results and table while ensuring that the discussion remains focused on the most relevant and statistically significant findings. However, we have refined the presentation of these results to highlight only the key differences that contribute meaningfully to the study's conclusions. Demographic comparisons have been reduced. ICU vs. medical ward patient satisfaction is analyzed properly. Regression assumptions have been addressed (normality test, VIF for multicollinearity).
**Discussion**	Provide a more in-depth discussion. Compare findings with other countries and consider the Palestinian healthcare system. Cultural influences were	The discussion now provides deeper comparisons with international studies and considers the Palestinian healthcare context. Cultural influences

	mentioned but not examined, clarify this.	are acknowledged as relevant but not directly examined in this study.
**Implications for Practice**	Add practical recommendations for nursing care improvement.	A new section on implications for practice has been added, including recommendations on nurse-patient communication, staffing, and patient-centered care.

Reviewer #2 – Point-by-Point (PBP) Response

Item	Comments	Response
**Sampling Method**	Clarify how the sample was selected.	Thank you for the suggestion. The sampling method is now clearly explained, including eligibility criteria and recruitment process.
**Social and Economic Factors**	The effects of social status, socio-economic factors, and education level on patient satisfaction were not analyzed.	Thank you for your valuable feedback. These factors were not included in the regression model, and their absence is now acknowledged as a study limitation. Future studies are recommended to explore their impact.
**Education Level and Satisfaction**	Does education level affect satisfaction? This was not addressed.	Thank you for the suggestion. Education level was not included in the analysis. This limitation is now acknowledged, and future research is encouraged to investigate its effect on patient satisfaction.
**Economic Status and Satisfaction**	Does economic situation affect satisfaction? This was not examined.	Thank you for your valuable feedback. Economic status was not analyzed in this study. This limitation has been added to the discussion, suggesting its inclusion in future research.
**Nurse-to-Patient Ratio**	Does the number of nurses per patient affect satisfaction?	Thank you for the suggestion. Nurse-to-patient ratio was not included in the study but is now recognized as an important factor affecting satisfaction. This is noted as a limitation, and future research should assess its impact.
**Nurses' Working Conditions**	The study does not consider the effect of nurses' working conditions on patient satisfaction.	Thank you for your valuable feedback. Working conditions (such as long shifts, burnout, and job satisfaction) were not analyzed but are now acknowledged as a limitation, with a recommendation for future research.
**Country's Health Policies**	What is the current situation of the country, and how does it impact health policies?	Thank you for the suggestion. The discussion now addresses how Palestine's healthcare challenges (staffing shortages, limited resources, and funding constraints) affect patient satisfaction and nursing care.
