# Supplementary material for: Patient satisfaction with the quality of nursing care in critical care units and medical wards in West Bank Hospitals, Palestine: A cross-sectional study
Source: PLoS One. 2025 Apr 29;20(4):e0322804. doi: 10.1371/journal.pone.0322804 (PMC12040242; doi:10.1371/journal.pone.0322804)
Supplement: Attachment — Submitted filename: Response_to_Reviewers_auresp_2.pdf [file pone.0322804.s004.pdf]

Point-by-Point (PBP) Table based on the provided Reviewer/Editor comments:

Reviewer/Editor	Comment Number	Item	Comment	Response
**Journal**	1	References	Ensure reference list completeness and correctness; address retracted papers if cited.	All references have been reviewed. No retracted papers were cited. The reference list is complete, accurate, and formatted per journal guidelines.
**Reviewer #1**	1	Abbreviations	Inconsistent abbreviation usage (e.g., "medical ward" vs. "MW"). Ensure abbreviations are defined once and used consistently.	Abbreviations (e.g., ICU, CCU, MW) are now defined at first mention and used consistently throughout the manuscript.
**Reviewer #1**	2	Title	Specify study design (Cross-sectional) and replace "ICU" with "Critical Care Units."	The title has been revised to: *"Patient Satisfaction with the Quality of Nursing Care in Critical Care Units and Medical Wards in West Bank Hospitals, Palestine: A Cross-sectional Study."*
**Reviewer #1**	3	Abstract	Add data collection duration, restructure results (use M/SD instead of range), and move implications to conclusion.	- Added data collection period: *"June–August 2024."*<br>- Results rephrased (e.g., *"M = 64.50, SD = 14.16"*).<br>- Conclusion now focuses on implications (e.g., targeted

				interventions).
**Reviewer #1**	4	Introduction	Discuss quality of nursing care gaps globally and locally. Clarify differences between critical care units and medical wards.	Expanded introduction to highlight international/local disparities in nursing care quality. Added a paragraph differentiating care intensity and patient interactions between critical care units and medical wards.
**Reviewer #1**	5	Methods	Clarify hospital selection criteria, inclusion/exclusion criteria, scoring system, and translation of the tool.	- Added: *"Four hospitals were selected to represent governmental/non-governmental sectors in urban/semi-urban areas."*<br>- Inclusion/exclusion criteria moved to a dedicated subsection.<br>- Clarified PSNCQQ scoring (19–95 scale; high score = high satisfaction).<br>- Specified that the English version was used (participants were proficient).
**Reviewer #1**	6	Results	Avoid redundancy; report only significant findings.	Non-significant results (e.g., marital status, room occupancy) were condensed or removed. Focus retained on predictors (age, health status) and ICU vs. ward differences.

**Reviewer #1**	7	Discussion	Provide deeper interpretation of results and link to global/local contexts.	Expanded discussion to compare findings with studies from Jordan, Egypt, and conflict-affected regions. Added policy implications (e.g., staffing ratios, training programs).
**Reviewer #1**	8	Language	Manuscript requires language editing for clarity and grammar.	The manuscript has undergone thorough language editing by a professional service to correct grammatical errors and improve readability.
**Reviewer #2**	1	General	Minor refinements needed before publication.	Addressed all minor edits (e.g., formatting consistency, typographical errors). Final proofreading completed.
